# Taking its TOLL: the role of toll-like receptor 4 in human health and disease, and its potential as a therapeutic target

**DOI:** 10.3389/fimmu.2026.1761361

**Published:** 2026-03-05

**Authors:** Phoebe Crammond, Priyanka Hastak, Anthony Delaney, Sarah C. Sasson

**Affiliations:** 1 Immunovirology and Pathogenesis Program, Kirby Institute, University of New South Wales, Sydney, NSW, Australia; 2 Intensive Care Unit, Royal North Shore Hospital, Sydney, NSW, Australia; 3 Critical Care Program, The George Institute for Global Health, University of New South Wales, Sydney, NSW, Australia

**Keywords:** damage associated molecular patterns (DAMPs), immunomodulating therapeutics, innate immunity, pathogen associated molecular patterns (MAMPs/PAMPs), TLR4, TLR4 agonists, TLR4 antagonists, toll-like receptor 4

## Abstract

Toll-like receptor 4 (TLR4) is a pattern recognition receptor that binds to pathogen associated molecular patterns (PAMPs) and damage associated molecular patterns (DAMPs). Binding of exogenous or endogenous ligands activates the TLR4 pathway and induces the production of pro-inflammatory cytokines TNF-α, IL-6 and IL-1β and type 1 interferons including IFN-α and IFN-β. TLR4 plays a vital role in host defense against Gram-negative bacterial infections by recognizing lipopolysaccharides (LPS) and inducing inflammatory mediators to clear infection. However, there is emerging evidence that excessive TLR4 activation may be pathogenic in human diseases affecting the central nervous system, cardiovascular, respiratory and metabolic systems, thereby promoting inflammation and autoimmunity. In some diseases, the is conflicting evidence regarding pathogenic versus protective roles. Several TLR4 targeted therapeutics have been developed and studied in animal models, however many of these therapeutics including Eritoran and TAK-242 did not prove to be effective in clinical trials. Overall, TLR4 exhibits context-dependent protective and pathogenic roles across infectious and non-infectious diseases, reflecting the complexity of its signaling in human health and disease.

## 1 Introduction

The activation of Toll-like receptors (TLRs) leads to the induction of a pro-inflammatory response which is important for defense against pathogenic infection. Toll-like receptor 4 (TLR4) activation which is classically involved in the defense against bacterial infections has been implicated in a variety of different diseases affecting several physiological systems. Furthermore, various TLR4-binding ligands have also been associated with disease pathology. Novel TLR4 therapeutics like Eritoran and TAK-242 have demonstrated efficacy in animal models of liver fibrosis, cardiac hypertrophy, myocardial ischemia-reperfusion injury, trauma, sepsis, post-operative abdominal adhesion (1–6). However, the efficacy of these therapeutics has not yet been translated to human clinical trials (5, 7, 8). TLR4-binding DAMPs may also be a potential therapeutic target for many of these diseases due to upregulated DAMP expression. Here, the role of TLR4 in human disease is reviewed, with key knowledge gaps highlighted. Further delineating the roles of TLR4 in human disease could pave the way for more efficacious therapeutic strategies for a variety of inflammatory diseases.

### 1.1 Toll-like receptors

Toll-like receptors (TLRs) are pattern recognition receptors (PRRs) which play a crucial role in the innate immune response. Human TLRs (TLR1 to TLR10) interact and bind to various pathogen associated molecular patterns (PAMPs) from viral and bacterial species, as well as alarmins or endogenous damage associated molecular patterns (DAMPs) including heat shock proteins (HSPs), heparan sulphate (HS) and high mobility group box protein 1 (HMGB1) (9–11). TLRs are predominantly expressed on or within innate cells including neutrophils, macrophages, monocytes, dendritic and epithelial cells (12). TLRs have also been characterized on innate lymphoid cells such as natural killer (NK) cells and are thought to activate these cells during microbial infection and initiate the first line of defense (13). Recent evidence suggests that TLRs can also be expressed on adaptive immune cells. Although generally low, TLR1–10 mRNA expression has been reported in human CD4^+^ and CD8^+^ T cells (14). Regulatory T cells (T_reg_) have also been shown to express TLR4,5,7 and 8 when challenged with LPS (15). Similarly, expression of TLR1, TLR6 and TLR10 mRNA has been identified in B cells (16).

### 1.2 Canonical toll-like receptor 4 signaling

**Figure 1:**
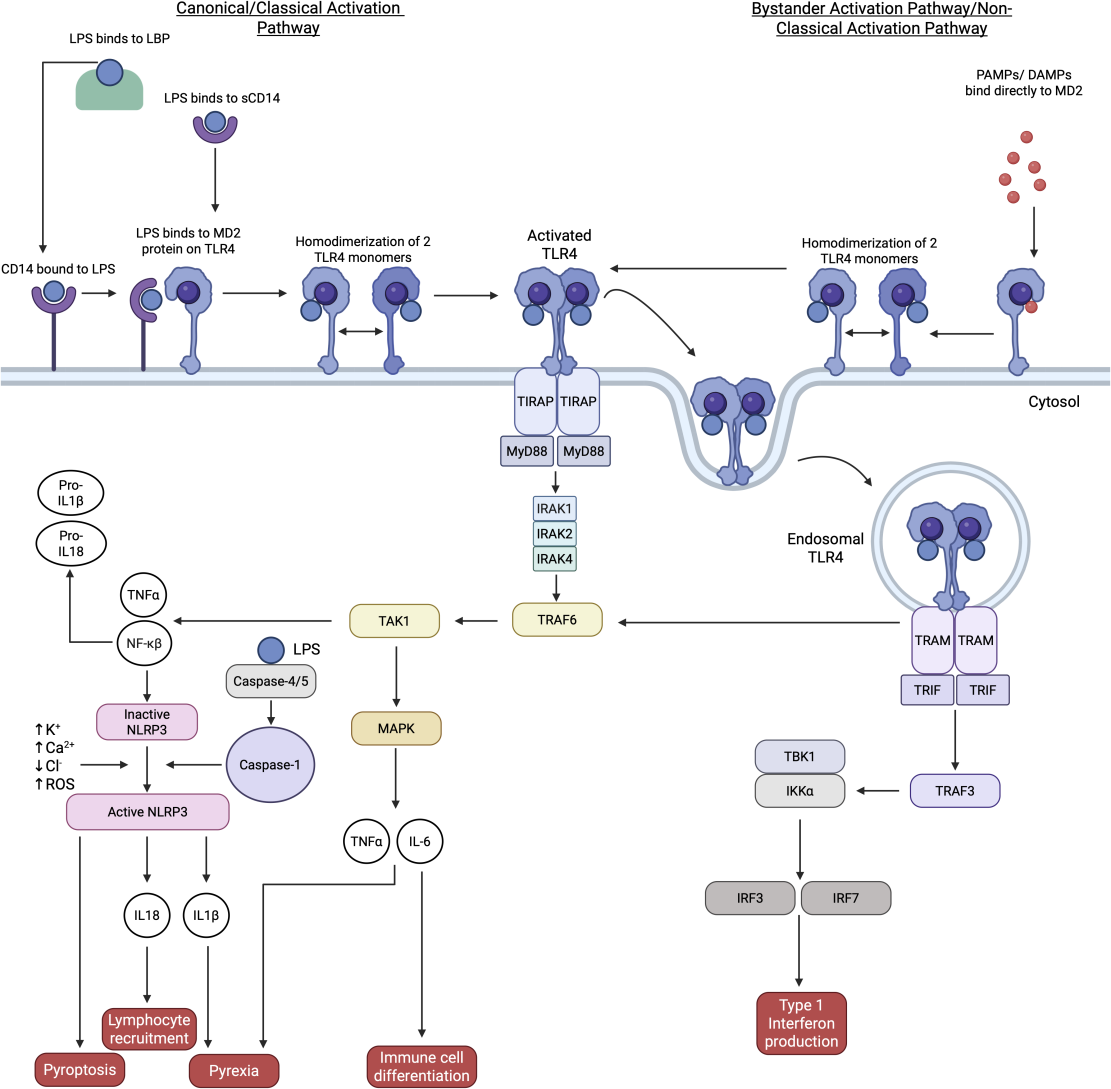
The TLR4 canonical and non-canonical pathways are shown. The canonical pathway occurs on monocytes through binding of LPS to LBB which binds to CD14, CD14 binds to the MD2 component of the TLR4 protein which stimulates homodimerization of two TLR4 monomers and activates the TLR4 protein which leads to a cascade of downstream molecule activation (18). The non-canonical pathway involves the activation of the TRIF/TRAM pathway independently of MyD88 activation and involves endocytosis of TLR4 (19, 20). Non-canonical activation of the NLRP3 inflammasome is also shown through activation of caspase-1 through caspase-4/5 (21, 22). Furthermore, the bystander activation pathway is described in this figure which involves non-classical binding of PAMPs and DAMPs as well as cytokines directly to the MD2 component of the TLR4 cell surface protein (namely T cells) (23). This binding leads to homodimerization of 2 TLR4 monomers which activates the TLR4 pathway. Activation of this pathway leads to pro-inflammatory mediators being expressed such as type 1 interferons, IL-6, TNF-α, IL-1β and IL-18 and induction of pyroptosis, lymphocyte recruitment, immune cell differentiation and type 1 interferon production (24). Activation of this pathway also drives NLRP3 inflammasome recruitment through caspase-1 activation (12, 18). Created in https://BioRender.com.

TLR4 was the first PRR discovered (initially denoted as *hToll*) and was identified in *Drosophila* and defined by its antimicrobial function (17). The canonical TLR4 signaling pathway refers to the activation of TLR4 on the surface of innate cells such as monocytes and subsequent MyD88 and TIR domain-containing adapter inducing IFN-β (TRIF) dependent pro-inflammatory cascade. TLR4 consists of three distinct domains including the cytoplasmic Toll-interleukin 1 receptor (TIR), leucine rich extracellular and transmembrane domains (12). Extracellular TLR4 proteins exist on the outer cell membrane as a monomer attached to Myeloid Differentiation factor 2 (MD2) when unbound to ligands (**Figure 1**). TLR4 has been repeatedly demonstrated to interact with the outer membrane component of Gram-negative bacteria, lipopolysaccharide (LPS) (12). Whilst non-classical binding of various DAMPs including HSP, hyaluronic acid (HA), HS, S100A4 and HMGB1 as well as other PAMPs like Severe acute respiratory syndrome coronavirus 2 (SARS-CoV-2) have also demonstrated to stimulate TLR4 activation (11, 25–28). The classical TLR4-LPS binding process is complex and requires various components. LPS binds to a lipopolysaccharide binding protein (LBP) which interacts with the CD14 receptor on the plasma membrane of monocytes. Once bound, the CD14 molecule dimerizes with TLR4 and binds LPS to the MD2 protein which is transported directly to the TLR4 binding site (**Figure 1**) (18, 19). Canonically, homodimerization of two TLR4 surface proteins occurs and recruitment of TIR domain containing adaptor protein (TIRAP) and MyD88 proteins transpires. TNF receptor associated factor 6 (TRAF6) self-ubiquitination leads to the activation of transforming growth factor-beta-activated kinase-1 (TAK1) and subsequent activation of the transcription factor nuclear factor-κβ (NF-κβ) (**Figure 1**) (29). NF-κβ transcription is key to the induction of inflammation through the production of pro interluekin-1beta (pro-IL-1β) and pro-IL-18 and initiation of the assembly of the NOD-, LRR-, and pyrin domain-containing protein 3 (NLRP3) inflammasome. Induction of caspase-1 and influx of potassium, calcium and reactive oxygen species (ROS) leads to the activation of the NLRP3 inflammasome and subsequent IL-18 and IL-1β production (12, 18, 19, 29). TAK1 activation also leads to the induction of other pro-inflammatory mediators such as IL-6 and TNF-α (12). In addition to the MyD88 pathway, the TRIF and TRIF related adaptor molecule (TRAM) are recruited and induce the production of type 1 interferons through TRAF3 activation (18).

### 1.3 Non-canonical toll-like receptor 4 signaling

Non-canonical TLR4 signaling refers to the activation of TLR4 and downstream products which is divergent from the recruitment of canonical TIRAP/MyD88 adaptor proteins and usually utilizes TRIF/TRAM signaling. For example, in murine dendritic cells, it has been shown that TRIF loss of function (LOF) resulted in impaired cytokine (e.g. IL-6, IL-12, IL-15, IL-18 and IL-23) and chemokine (e.g. CXCL10 and CCL12) expression when compared to wild type (WT) cells (30). This finding indicates that production of these mediators is dependent on a functional TRIF adaptor protein. Furthermore, a recent study suggests that ubiquitin mediates a TIR dependent process that drives TLR4 endocytosis, however this process is independent of recruitment of adaptor proteins which are vital to canonical TLR4 pathway activation (20). Interestingly, caspase-4/5 has been shown to recruit caspase-1, facilitate potassium and calcium efflux and stimulate Gasdermin D pore formation which activates the NLRP3 inflammasome. This caspase-4/5 induced NLRP3 activation is thought to be through direct detection of intracellular LPS, independent of TLR4 or TNFα receptor activation (**Figure 1**) (21, 22). Therefore, NLRP3 can also be activated independently of TLR4 activation.

### 1.4 The bystander activation pathway

This article highlights several human diseases which are ‘sterile’ (i.e. non-infectious) in nature, where TLR4 activation occurs in the absence of LPS-TLR4 dimerization to initiate TLR4 activation. More recently the TLR4 bystander activation pathway has been described, which is divergent from the classical TLR4 signaling pathway on monocytes. The bystander activation pathway proposes an alternative way of activating the TLR4 pathway through cytokines, paracrine signaling and DAMP’s, without the need of classical LPS binding (**Figure 1**).The bystander activation pathway has been proposed on a variety of cells, including on T lymphocytes without canonical T cell receptor (TCR) activation (23). This pathway was first proposed on memory CD8^+^ T cells in 1996 where CD8^+^ T cell clonal expansion in systemic viral infections occurred independently of TCR activation (31). It has been shown that the TLR4 pathway occurs in “innate-like” memory and naïve CD8^+^ T cells in sterile inflammation (32). Although less investigated, effector CD4^+^ T cells have also demonstrated to change to a less inflammatory phenotype when challenged with LPS, independent of TCR activation (33).

However conflicting evidence suggests that CD4^+^ T cell activation from LPS requires crosstalk between TLR4 and TCR with macrophage inhibitory factor (MIF), a cytokine secreted by activated T cells that impedes the migration of macrophages, regulating TLR4 expression on CD4^+^ T cells (34). As previously mentioned, TLR4 classically recognizes and directly binds to LPS through LBP and CD14 on the plasma membrane of innate cells. However, PAMPs and DAMPs have been shown to bind to the MD2 component directly, without the need for canonical LBP or CD14 co-receptor binding through the bystander pathway, providing further evidence of TLR4 activation which is not classical in nature (23, 35, 36).

## 2 Therapeutic targeting of TLR4

### 2.1 TLR4 agonists

Agonists against various TLRs have been licensed for clinical use for the treatment of bladder cancer, basal cell carcinomas and genital warts (37–39). Agonists that specifically target TLR4 have been successfully incorporated into vaccines to enhance efficacy and have been further investigated in clinical trials for breast cancer, sarcomas and other advanced solid tumors due to their ability to induce pro-inflammatory mediators which encourage tumor destruction. The main TLR agonists which are in current clinical use are outlined below.

#### 2.1.1 Bacillus Calmette-Guerin vaccine

The Bacillus Calmette-Guerin (BCG) vaccine, designed to prevent *M. tuberculosis* infection, is a well-established immunomodulating agent which is commonly used for the treatment for superficial bladder cancers (40). The mechanism of action for the BCG vaccine is thought to be mediated through the TLR4 pathway which leads the production of TNF-α, IL-6, and IL-1β shortly after vaccine administration, as well as downstream gene expression of inhibitor of nuclear factor kappa B kinase subunit beta (IKBKB), intracellular adhesion molecule 1 (ICAM-1) and C-C motif chemokine ligand 2 (CCL2) which are crucial for NF-κβ pathway activation (37, 40). Stimulation of the NF-κβ pathway after BCG vaccine administration leads to the activation of innate cells such as macrophages, granulocytes, dendritic cells and NK cells, but has also been shown to activate adaptive cells like CD4^+^ and CD8^+^ lymphocytes which drives the destruction of tumor cells (41).

#### 2.1.2 Monophosphoryl lipid A

Monophosphoryl lipid A (MPL) is a non-toxic and potent TLR4 agonist which is derived from LPS and has been widely utilized as a vaccine adjuvant. AS04 which combines MPL and aluminum salts with viral antigens, has been used as an adjuvant in the HPV bivalent (e.g. Cervarix^™^) vaccine, as well as the Hepatitis B DNA vaccine (e.g. Fendrix^®^), by promoting a more robust immune through the TLR4 and NLRP3 inflammasome pathways (42–44). The Herpes zoster vaccine (e.g. Shingrix^™^) is a AS01_B_ vaccine which combines MPL with cholesterol derived liposomes and the saponin QS21, that has been widely adopted for the prevention of shingles (45).

#### 2.1.3 Glucopyranosyl lipid A in a stable-emulsion formulation

Glucopyranosyl lipid A in a stable-emulsion formulation (GLA-SE) is a potent TLR4 agonist which has been used in clinical trials as a vaccine adjuvant for infectious diseases including *M. tuberculosis* and *Plasmodium* infections causing Malaria (46, 47). Furthermore, an open label Phase I non-randomized clinical trial (RCT) of 12 adult (>18 years old) patients with palpable soft tissue sarcomas found that local GLA-SE administration in conjunction with radiotherapy reduced lesions. After 8 weeks of weekly intratumoral injections of GLA-SE (5μg-10μg) concurrent with radiotherapy, patients exhibited an average 69% decrease in tumor size and increase in local infiltrating T lymphocyte populations when compared to patients who received radiotherapy alone (48).

#### 2.1.4 GSK1795091

GSK1795091 is a selective TLR4 agonist and vaccine adjuvant which has demonstrated to elicit a strong protective response against a split influenza antigen [A/Uruguay/716/2007 (H3N2)] and promote the production of antigen specific Th17 cells through intranasal administration in mouse models (49). Furthermore in 2020, a Phase I double-blind RCT demonstrated that intravenous administration of GSK1795091 was well-tolerated in healthy volunteers with no serious adverse effects reported (50). In a Phase I open label non-RCT of 54 adult patients with advanced solid tumors, intravenous GSK1795091 (50-250ng) was administered in combination with anti-PD-1 (200mg), anti-OX40 (24mg) or anti-ICOS (80mg). Although a third of these patients reached disease stability, 94% experienced at least one adverse effect with the most common being chills, nausea and pyrexia (51). However, due to the small sample size, lack of control group and change in drug formulation, definitive conclusions could not be made regarding the efficacy of this adjuvant in patients with advanced solid tumors (51).

### 2.2 TLR4 antagonists

Multiple TLR antagonists have shown efficacy in clinical trials for the treatment of diseases including systemic lupus erythematosus (TLR7/8 antagonist) and psoriasis (TLR7/8/9 antagonist) (52, 53). However currently, there are no TLR antagonists licensed for clinical use. Candidate TLR4 antagonists have been developed given that TLR4 has been implicated in a wide variety of inflammatory diseases affecting the central nervous system (CNS), respiratory, cardiovascular, gastrointestinal and metabolic systems in both animal and human studies. These candidate TLR4 antagonists have been tested preclinically and in early-stage clinical trials as outlined below.

#### 2.2.1 Eritoran

Eritoran is a synthetic lipid A analog that binds to the MD2 protein that is recruited by TLR4 after homodimerization and inhibits TLR4/MD2 signaling. In animal models, Eritoran ameliorates excessive pro-inflammatory responses in liver fibrosis, cardiac hypertrophy, myocardial ischemia-reperfusion injury, trauma and sepsis, indicating a potential role for TLR4 in these disease processes and the potential for therapeutic modulation (1–4). However, these findings have not clearly translated in human clinical trials of Eritoran. In a Phase III double-blind RCT conducted in 1961 adult patients with sepsis, Eritoran did not reduce mortality when compared to the placebo group. Eritoran tetrasodium (6.56 - 26.24mg) was administered serially every 12 hours to septic patients within 12 hours of organ dysfunction onset over a period of 6 days but did not lead to a significant reduction in the 28-day mortality in the Eritoran group when compared to placebo (5). Additionally, Eritoran has been explored in settings other than sepsis, for example in obesity related insulin resistance. A clinical trial of nine patients with a body mass index (BMI) of 30-37, indicated that administration of Eritoran did not confer a protective role against obesity-induced insulin resistance (7).

#### 2.2.2 TAK-242

Similar to Eritoran, TAK-242 is a TLR4 antagonist which has shown promise in preventing disease in animal models but has not demonstrated efficacy in humans. In mouse models, a combination of both TAK-242 and sodium hyaluronate (HA) reduced localized reactive oxygen species and alleviated collagen deposits in postoperative abdominal adhesions (5). However, in a RCT of 274 septic patients, administration of TAK-242 (1.2mg/kg) within 36 hours of disease onset followed by a continuous infusion for up to 120 hours (1.2mg/kg - 2.4mg/kg per day) failed to reduce increased cytokine (e.g. IL-6) expression or improve 28-day mortality when compared the placebo group (8).

#### 2.2.3 FP7 & FP12

FP7 is a di-phosphorylated monosaccharide which acts as a TLR4 antagonist. It has been shown to effectively inhibit increased TLR4 expression following LPS stimulation, as well as improve survival in mouse models of severe influenza and sepsis (54, 55). FP7 has also shown to attenuate inflammation in vascular tissue through the inhibition of TLR4, however to-date FP7 has not progressed to human clinical trials (56).

FP12 is a more recently developed synthetic glycolipid-based TLR4 antagonist that has been shown to ameliorate severe influenza infection in mice models by reducing lung inflammation and HMGB1 expression; but again, has not progressed to human clinical trials (57).

#### 2.2.4 JKB-122

JKB-122 is a weak TLR4 antagonist that has shown efficacy in pre-clinical trials. In mice, combination treatment of JKB-122 with prednisolone reduced liver lesions and TLR-4 associated-inflammation in autoimmune hepatitis (58). Furthermore, a Phase II clinical trial (NCT02556372) of patients with autoimmune hepatitis treated with JKB-122 demonstrated a reduction in alanine aminotransferase, however further results from this study are pending (59, 60).

#### 2.2.5 CRX-526

CRX-526 is a competitive TLR4 antagonist which has not been widely used. CRX-526 has been shown to ameliorate renal destruction in mouse models of diabetic nephropathy and prevent the development of severe disease in murine inflammatory bowel disease (IBD) (61, 62). However, like most of the other TLR4 antagonists listed above, no human trials have been conducted.

## 3 The role of TLR4 in disease

**Figure 2:**
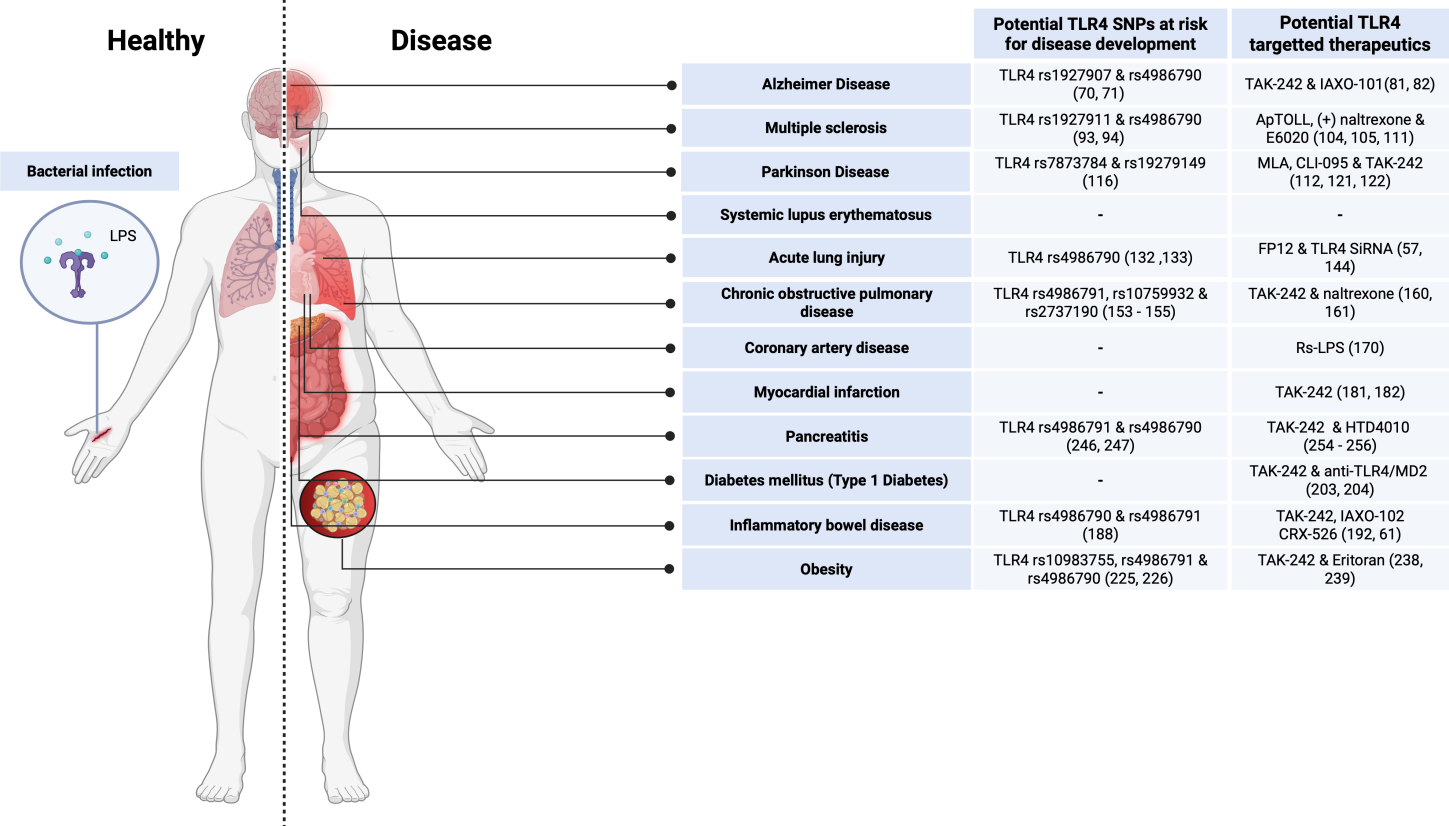
Known roles of TLR4 signaling in human health and disease. The right side of the table outlines diseases currently associated with TLR4 genetic variant association, as well as potential TLR4 targeted therapeutics that have been used in animal models for specific inflammatory diseases. Created in https://BioRender.com.

TLR4 has an ameliorating effect during bacterial and viral infections by inducing pro-inflammatory mediators. However, when TLR4 activation is dysregulated, inflammatory diseases can occur (**Figure 2**).

### 3.1 Central nervous system diseases

#### 3.1.1 Alzheimer disease

Alzheimer disease (AD) is a progressive neurodegenerative disease with rising prevalence in aging populations. It is defined by the accumulation of amyloid beta (Aβ) plaques leading to atrophy in the hippocampus, neuronal loss and cognitive decline (63). Memantine, a N-methyl-D-aspartate (NMDA) receptor antagonist and cholinesterase inhibitors are symptom-focused therapeutics which are commonly used to treat AD (64, 65). More recently, disease modifying drugs such as Lecanemab and Donanemab which focus on modifying AD pathogenies to reduce disease severity have been introduced to treat early AD (66, 67). Although AD is mainly characterized by the build-up of Aβ plaques, there is emerging evidence of other markers, including TLR4 being associated with the pathology of AD.

Many AD cases are sporadic, however the main non-modifiable risk factors for AD include older age, family history and female biological sex; whilst modifiable risk factors include excessive alcohol intake, smoking, obesity, poor sleeping patterns, low social interaction and physical inactivity (68, 69). Moreover, there is evidence that TLR4 genetic variants may contribute to AD risk. Studies have linked TLR4 dysfunction with several AD risk factors, including cardiovascular disease, obesity and diabetes to be outlined further below. Polymerase chain reaction (PCR) analysis of DNA from patient blood samples and genotyping of TLR4 single nucleotide polymorphisms (SNPs) identified TLR4 polymorphisms that may increase or decrease the risk of AD in in a variety of global populations. For example, in Northern Taiwan the TLR4 SNP rs1927907 was associated with increased risk of late onset AD (LOAD) in 269 patients with LOAD compared to 449 elderly healthy controls recruited at health check-ups, all ≥ 65 years old (70). Additionally, a study conducted in China, demonstrated in a cohort of 274 combined late onset AD patients and age/sex-matched healthy controls, the minor allele of the TLR4 rs4986790 polymorphism (C allele) was associated with an almost 6-fold risk of developing late onset AD when compared to the G allele (71). Comparably, a separate study identified from 136 participants from the PREVENT-AD cohort in Canada, that the G allele of the TLR4 rs4986790 SNP was associated with a decreased risk of LOAD by modulating the production of IL-1β and decreasing amyloidosis when compared to age-matched controls (72). Similarly, a study conducted in Italy found that the G allele of the TLR4 rs4986790 SNP was associated with a decreased risk of LOAD when comparing the presence of this allele in 277 LOAD patients to 300 age and sex-matched controls (73).

Some studies suggest TLR4 plays a pathogenic role in AD. In a post-mortem study, TLR4 and TNF-α mRNA levels were significantly higher in the frontal cortex of patients with AD as compared to age-matched controls (74). Additionally, flow cytometry and real-time quantitative polymerous chain reaction (RT-qPCR) of peripheral blood mononuclear cells (PBMCs) showed that TLR4 expression was significantly higher in LOAD patients when compared with healthy sex and age-matched controls (75). Given that obtaining brain biopsies from AD patients presents a difficult challenge, much of the work investigating the effect of TLR4 on AD has been conducted in mouse models. In TLR4 and TLR2 knockout (KO) mice, TLR4 and co-receptors CD14 and TLR2 were necessary to activate phenotypic class switching of microglial cells which play a critical role in inducing phagocytosis of A*β* plaques and production of destructive reactive oxygen species (ROS) (76). Microglial cells are resident immune cells of the CNS and in healthy people these cells act to clean up debris. However, in AD these cells display some autoreactive functions, as they can stimulate phagocytosis of A*β* plaques but also drive A*β* plaque production which leads to neurodegeneration (77). The pathogenic role of TLR4 is further supported by another study that found in mouse models of AD there was an increase of TLR4 expression on microglial cells (CD11c^+^ Clec7a^+^) (78). Furthermore, this study also demonstrated in TLR4 KO/WT mice that Lyn kinase (Lyn) is a non-canonical TLR4-binding intracellular adaptor protein which is associated with AD pathogenesis. When Lyn was absent there was a reduction in neuronal atrophy and increased Aβ plaque clearing (78). When Lyn was present in the AD mice models, there was an increase of pro-inflammatory cytokines, indicating that this kinase may play a role in regulating homeostasis through TLR4 expression (78). Another study observed heightened TLR4-Lyn expression on microglial cells and cognitive decline in female mice when compared to male mice, indicating that females may have an increased risk of AD (79).This is also demonstrated by another study in male mice, where the hormone dihydrotestosterone reduced TLR4-mediated cytokines in serum and brain following LPS stimulation (80). Therefore, this combined evidence of heightened TLR4-Lyn expression in female mice and reduced TLR4-mediated inflammation post dihydrotestosterone administration may provide some insight into why AD risk is increased in women compared to men. TLR4 modulating therapies could provide benefit to patients with heightened TLR4 expression in AD with studies suggest that inhibition of TLR4 may provide neuroprotective properties. In mouse models, TLR4 antagonists TAK-242 and IAXO-101 promoted neuroprotection by reducing autoreactive microglial cells (81, 82). Yet, these therapeutics have not been translated to clinical trials for AD. Various well-established and lesser known TLR4-binding ligands have shown to be associated with AD pathogenesis. Through an *in vitro* immortalized mouse cell line, it has been shown that a specific A*β* protein known as A*β*_1–42_ can bind directly to TLR4 as an endogenous ligand and stimulate microglial cell activation (83). Furthermore, more well-established ligands including HMGB1, HA, HSP and HS have all been implicated in AD pathogenesis (84–87). One study demonstrated that HMGB1 is increased in the cerebral spinal fluid of patients with AD when compared to age and sex matched healthy controls. This study also demonstrated that subcutaneous injection of anti-HMGB1 reduced DNA damage significantly in the cortex of AD-induced mice and reduced Aβ plaques (84). Therefore, TLR4-binding ligand specific therapeutics may also be efficacious in reducing AD symptoms and disease manifestation.

Some contrasting studies suggest that TLR4 may ameliorate AD. An AD mouse model characterized by TLR4 LOF found that TLR4 was not responsible for A*β* plaque deposits but instead promoted A*β* plaque clearance through activation of microglial cells to drive phagocytosis and increase cognitive function (88). TLR4 targeted therapeutics may therefore represent potential disease modifying agents in AD with one study showed that stimulating microglial cells with low-level LPS in rats exerted neuroprotective affects (89). Therefore, low level stimulation of TLR4 could be protective, whereas chronic or excessive expression could lead to AD manifestation.

The role of TLR4 in AD seems to be largely pathogenic. When activated appropriately, TLR4 may exhibit neuroprotective properties (88). However, when there is excessive or dysfunctional TLR4 activation due to a predisposing genetic variant and risk factors inducing chronic inflammation, disease such as AD may manifest (70, 71, 79, 80). TLR4 or TLR4-binding ligands such as HMGB1 targeted therapeutics may therefore be useful in reducing neuroinflammation mediated by chronic TLR4 activation, however further delineation of the exact function of TLR4 in AD is required.

#### 3.1.2 Multiple sclerosis

Multiple sclerosis (MS) is an autoimmune disease of the CNS characterized by the demyelination of neurons, which is driven by autoreactive innate and adaptive cells. Current disease modifying agents in clinical use for MS include B-cell depleting monoclonal antibodies and interferon beta (IFNβ) (90–92). As MS is a heterogenous disease in which therapeutic efficacy varies from patient to patient, additional targeted therapeutics is an area of ongoing need.

There is ongoing research into the genetic determinants of MS, with recent articles suggesting TLR4 polymorphisms could increase risk. In a 2024 study in Iran, the TLR4 rs1927911 SNP with the C/C and T/C genotypes were more highly expressed in 200 MS patients with primary progressive (PP), secondary progressive (SP) and relapsing-remitting (RR) MS when compared to 200 matched healthy volunteers (93). An earlier study found that PBMC proliferation was reduced in 6 healthy controls with the TLR4 rs4986790 SNP with the A/G genotype post LPS stimulation when compared to 6 controls with the WT A/A genotype (94). Further investigation into TLR4 SNPs in MS is therefore required to determine a genetic dysfunction on this receptor. More recently, Epstein-Barr Virus (EBV), the herpesvirus that leads to mononucleosis infection, has been heavily associated with MS development, with over 900 patients after EBV infection demonstrating an approximate 30-fold elevated risk of developing MS (95, 96). A study which investigated almost 150 young people (<18 years old) with active EBV infection found the TLR4 rs4986790 SNP was associated with EBV development when compared to WT allele (97). This data highlights a potential link between EBV infection and the development of MS, that may be facilitated or amplified by TLR4 SNPs such as TLR4 rs4986790.

The exact role of TLR4 in MS remains unclear, however it has been postulated to be pathogenic. The Multiple Sclerosis-associated Retrovirus (MSRV) on activated microglial cells of patients with MS has been demonstrated to be a competitive agonist of TLR4. Like LPS, binding of TLR4 by MSRV induces downstream pro-inflammatory cytokines like IL-6 and TNF-α (98). Therefore, TLR4 could contribute to the pathogenesis of MS through direct MSRV binding. TLR4 protein expression was found to be significantly higher on lymphocytes, neutrophils and extracellular vesicles (EV) in MS patients, when compared to matched controls (99, 100). However, other studies have demonstrated a reduction in TLR4 expression in MS patients. Some studies showed that TLR4 protein and mRNA expression on monocytes was reduced in RR-MS patients when compared with healthy controls, suggesting that a lack of TLR4 expression could drive MS pathogenesis (101, 102). As MS is currently understood to be predominantly driven by autoreactive immune cells, the expression of TLR4 on immune cells may provide insight to the induction of autoreactivity. Additionally in murine cell lines of MS, the endogenous DAMP HMGB1 was increased compared to controls and was linked to NLRP3 mediated pyroptosis as well as neuronal cell death *in vitro (*103). As HMGB1 is a TLR4 ligand, targeting this pathway is of potential interest for future research. If excessive TLR4 expression contributes to MS disease severity, then modulating this pathway with agents targeting the TLR4 binding site may be an effective therapeutic avenue. Modulating the TLR4 pathway has shown to exhibit some therapeutic effects in MS. For instance, a study that investigated the effect of ApTOLL, a selective TLR4 antagonist, in mouse models and primary human cell cultures, showed that this antagonist promoted remyelination as well as oligodendrocyte precursor cell production (104). Furthermore, a TLR2/TLR4 antagonist called (+) naltrexone (a positive isomer of the opioid antagonist naltrexone) reduced the neuroinflammatory phenotype in the hippocampus in mouse models of MS (105). Various TLR4-binding ligands have been associated with MS disease progression and may also be potential therapeutic targets. In an MS mouse model, HA has been shown to drive MS disease progression by T-helper cell 1 (Th1) polarization resulting in auto-reactive T cell production (106). Blocking HA production with 4-methylumbelliferone (4-MU) (a competitive antagonist for UDP-glucuronyltransferase which is involved in HA synthesis) in mice has demonstrated efficacy by reducing spinal cord lesions and inducing Foxp3+regulatory T cells (T_regs_) which resulted in a reduced auto-reactive phenotype (106, 107). Furthermore, a meta-analysis of 364 MS patients showed a significant increase of HMGB1 protein on PBMCs and in the CSF when compared to controls (108). Therefore, HMGB1 could also be explored as a potential therapeutic target for MS.

There is contrasting data to suggest the role of TLR4 in MS may also have protective effects. *In vitro* concurrent TLR4 and CD40 receptor stimulation has been shown to stimulate the production of intracellular IL-10 in RR-MS PBMCs when compared to sex-matched healthy controls (109). This data suggests the role of TLR4 in MS is multifactorial as IL-10 provides anti-inflammatory effects which assists in decreasing neuroinflammation. Another study found that the efficacy of IFNβ treatment in MS patients could be mediated through the TLR4 pathway as IL-10 was significantly higher in LPS stimulated whole blood cultures of patients treated with IFNβ when compared to healthy controls stimulated with LPS (110). Interestingly, in a rat model of AD, the TLR4 agonist E6020 promoted remyelination of white matter and accelerated phagocytosis to clear myelin debris, supporting the hypothesis that TLR4 expression drives neuroprotective effects (111). This treatment is contradictory to the previously discussed studies that suggest a pathogenic effect of TLR4 in MS (98–100, 103–105). This may be because of differing models that were used, such as a rat model for the investigation of E6020 whereas ApTOLL and (+) naltrexone were used in mouse models and primary human cell cultures (104, 105, 111). Therefore, a comparison between these models should be considered in the future when investigating the effects of TLR4 targeted therapeutics in MS. Data delineating the exact role of TLR4 in patients with MS is lacking, despite evidence of TLR4 genetic variant association and overall TLR4 involvement in this disease. Further investigation is required to determine appropriate TLR4 therapeutic targeting.

#### 3.1.3 Parkinson’s disease

Parkinson’s disease (PD) is a neurodegenerative disease defined by the accumulation of misfolded Alpha-synuclein (α-Syn) protein aggregations in the substantia nigra and subsequent dopaminergic neuron destruction (112, 113). The current gold standard treatment for PD is the dopamine-precursor drug LevaDopa which was founded over 60 years ago (114). There are few other therapeutic options available for this disease, highlighting a need for further research.

Available evidence suggests that TLR4 SNPs may be involved in PD, with studies predominantly from China identifying TLR4 polymorphisms associated with PD development. The TLR4 rs1927914 C allele polymorphism has been associated with reduced risk of PD in a study of genomic DNA from PBMC’s isolated from 380 PD patients compared to 380 matched healthy controls (115). Furthermore, when investigating almost 1,300 combined PD and control patients, carriers for the TLR4 rs7873784 G allele or the TLR4 rs19279149 C allele had a significantly higher risk of PD development compared to those with WT genes (116). Although these studies may highlight a link between certain TLR4 polymorphisms and PD, further investigation is required to identify prevalent polymorphisms in PD in a wider population.

TLR4 has been postulated to play a role in the pathogenesis of PD. In a study of over 200 participants, TLR4 protein from PBMCs and circulating HMGB1 was highly expressed in patients with PD compared to healthy controls and positively correlated to disease severity and progression (117). However, in a model where PBMCs from patients with PD underwent TLR4 stimulation through LPS, it was shown that downstream TNF-α was higher in young patients (≤ 55 years old) when compared to elderly patients (≥ 65 years old) and age-matched healthy controls (118). This suggests that older patients with PD have a dampened TLR4 activation response and suggests that TLR4 hyperresponsiveness could be more important in the earlier stages of PD, with immune tolerance occurring later in PD progression. Furthermore, prothrombin kringle-2 (pKr-2) which is a domain of the prothrombin protein that is released during vascular injury, has been associated with death of dopaminergic neurons. It has been suggested that pKr-2 is mediated through endogenous TLR4 binding on activated microglial cells in the substantia nigra in four PD patients when compared to four age-matched controls (113). Additionally, activated microglial cells were reduced in TLR4 KO mouse models of PD when compared to WT controls, further suggesting that increased TLR4 expression drives microglial activation and neuroinflammation in PD (113). Chronic exposure to low levels of α-Syn can sensitize TLR4 and lead to TLR4 hyperresponsiveness on neuronal cells, implying that protein aggregation, which is fundamental in PD, leads to TLR4 sensitization and excessive TLR4 upregulation, ultimately propagating an excessive proinflammatory response (119). On balance this data suggests that TLR4 inhibition may be useful in reducing neuroinflammation in PD. TLR4 KO mice injected with 1-methy-4-phenyl-1,2,3,6-tetrahydropyridine (MPTP) which is a neurotoxin used to model PD, demonstrated a decrease in TRAF6 and NLRP3 inflammasome expression after MPTP stimulation and subsequently prevented dopaminergic neuron degeneration, suggesting that TLR4 inhibition could provide therapeutic effects in PD (120). Furthermore, TLR4 and Dendritic cell-associated C-type lectin-1 (Dectin-1) crosstalk may contribute to PD severity. TLR4 induction promotes the activation of Dectin-1 on microglial cells in mouse models. The inactivation of Dectin-1 alleviates neurodegeneration by switching microglial cells from the proinflammatory M1 to anti-inflammatory M2 phenotype. Inactivation of TLR4 through the TLR4 antagonist CLI-095 subsequently inhibited Dectin-1 and promoted M1 to M2 microglial cell class switching, consistent with a more neuroprotective effect (121). Therefore, TLR4 inhibition may promote microglial phenotypic changes which evoke a neuroprotective effect in PD. Similarly, rats treated with 6-OHDA which models PD through dopaminergic neuron destruction, were stimulated with TLR4 antagonist TAK-242 and insulin or TAK-242 alone. The combination of TAK-242 and insulin exerted greater improvements in motor performance when compared to TAK-242 alone, due to the combination treatment targeting insulin resistance which is a hallmark of PD, and contributes to TLR4 downregulation (122). Multiple TLR4-binding ligands have also been associated with PD pathogenesis. A zebrafish model of PD showed that after MPTP administration, HMGB1 mRNA was significantly upregulated in the brains of zebrafish (123). Elevated HMGB1 has also been detected in human nigral tissue of deceased PD patients when compared to controls. The same study also found that anti-HMGB1 administration in a PD mouse model reduced dopaminergic neuronal death (124). Additionally, the knockout of genes encoding for HS in *Caenorhabditis elegans* reduced neurodegeneration and α-Syn aggregation, highlighting the crucial role for HS in PD (125). Therefore, targeting HMGB1 and HS may also represent potential avenues for new therapeutics to reduce PD disease progression.

Although several articles suggest TLR4 signaling exacerbates PD disease severity, there is conflicting evidence that TLR4 may also drive neuroprotection. A study of murine PD with the selective TLR4 agonist Monophosphoryl A (MLA) demonstrated prevention of dopaminergic neuron damage, as well as Alpha-synuclein clearance by activated microglial cells and consequently a significant improvement in motor activity (112). This indicates that in certain circumstances, TLR4 signaling could facilitate an ameliorating response in PD, potentially in elderly populations due to a dampened TLR4 signaling.

Overall, the role of TLR4 in neurodegenerative diseases remains controversial, with some studies suggesting TLR4 may drive neurodegeneration. For example, in AD, TLR4 may assist in driving autoreactive microglial cell production, and in PD, protein aggregation may lead to TLR4 sensitization which promotes proinflammatory mediators (76, 119). Therefore, TLR4 inhibitors may pose as an effective therapeutic in these circumstances. In contrast, other studies have found TLR4 signaling prevents disease progression. For instance, in MS, TLR4 and CD40 cross signaling may stimulate IL-10 production which induces an anti-inflammatory effect and in AD, TLR4 may promote αβ plaque clearance (88, 109). Further research is needed to understand in what circumstances TLR4 antagonists and agonists may be best deployed.

### 3.2 Respiratory diseases

#### 3.2.1 Acute lung injury

Acute lung injuries (ALIs) comprise a broad spectrum of respiratory related damage that are characterized by excessive infiltration of immune cells to the lungs. ALI can be subcategorized into direct and indirect ALI. Direct ALI occurs due to local injury to the lungs such as bacterial or viral community acquired pneumonia (CAP), whereas indirect ALI arises from distant inflammation such as pancreatitis, burns and non-thoracic trauma (126). Currently the most severe forms of ALI are managed in intensive care units (ICUs) with endotracheal intubation, mechanical ventilation, and antimicrobial drugs for infective ALIs (127). During the coronavirus disease 2019 (COVID-19) pandemic, the management for ALIs expanded to immunomodulating therapeutics including dexamethasone, baricitinib and tocilizumab, which were effective in reducing the severity of COVID-19-induced ALI (128–130). While the benefits of immunomodulation have been demonstrated in the ALI caused by COVID-19, the role of host-directed therapies in the settings of other forms of ALI is a frontier of current research.

The link between TLR4 polymorphisms in ALI have been investigated for virally induced ALI such as COVID-19. The GG/AG genotypes for the TLR4 rs4986790 SNP has been associated with protection against hospitalization in a study of 1,570 European patients with COVID-19 (131). Conversely, an earlier study of almost 1,200 hospitalized COVID-19 patients in Mexico suggested that the G/G genotype in the TLR4 rs4986790 SNP was associated with an increased risk of COVID-19 (132). Similarly, in a case control study of over 450 patients with COVID-19 in Morocco, the presence of the A/G genotype in the TLR4 rs4986790 SNP positively correlated with increased serum ferritin in severe COVID-19 patients (133). This contradictory data could be due to geographical heterogeneity as well as differing sample sizes. Furthermore, one study identified that SNPs for genes such as MyD88 rs7744, TNF-α rs1800629 and IL-6 rs1800796 which are typically upregulated after TLR4 activation, increased the likelihood of ALI development in 300 patients with direct or indirect ALI in China when compared to 300 matched ICU controls who did not have ALI (134). Although studies have investigated TLR4 SNPs in virally induced ALI, limited research has been conducted into TLR4 SNPs in other forms of direct ALI (e.g. bacterial induced ALI) and indirect ALI (e.g. severe trauma, burns and pancreatitis).

Increased TLR4 expression and related inflammation has been associated with ALI. One cross-sectional study showed that in almost 100 patients with COVID-19, soluble TLR4 (sTLR4) and CD14 (sCD14) were significantly higher in the patients with severe disease as compared to those with non-severe disease (135). This may indicate that there is an endogenous mechanism to regulate excessive TLR4 activation in severe COVID-19 as sTLR4 has been suggested to act as a negative regulator for TLR4 activation (135, 136). Interestingly, TLR4 upregulation has also been identified in non-infective ALI. A study which investigated 22 patients undergoing orthotopic liver transplantation, found that patients who developed ALI from reperfusion injury had a significantly increased level of TLR4 expression when compared to patients who did not develop ALI (137). Furthermore, in mouse models of hemorrhagic-shock-induced ALI, HMGB1 activates TLR4 which leads to the upregulation of co-receptor TLR2 on endothelial cells and initiation of a systemic pro-inflammatory response which migrates to the lungs (138, 139). Another study suggested that after hemorrhagic shock, HMGB1 binding to TLR4 in the intestinal tract leads to ALI. This was demonstrated through mice which lacked TLR4 expression in the intestinal epithelium (TLR4^ΔIEC^ mice) and which were placed into hemorrhagic shock. A protective response against ALI was evident as well as lowered circulating HMGB1 levels when compared to WT mice (140). This data suggests that hemorrhagic-shock-induced ALI is reliant on TLR4 activation through HGMB1 accumulation in the gut and subsequent pro-inflammatory cell release to the lungs. Although, this pathway has not been fully delineated, further investigation into TLR4 expression in the lung-gut axis could provide insight into hemorrhagic shock-induced ALI and other ALIs involving the gastrointestinal tract. Additionally, treating mice with intratracheal LPS has been shown to activate the NLRP3 inflammasome through the TLR4 pathway and subsequently upregulate IL-1R expression on alveolar macrophages (AM), allowing for sensitization and pyroptosis of AMs which exacerbates lung inflammation (141). Furthermore, TLR4 KO mice exhibited protection from ALI when compared to the WT controls, by preventing pathomorphological changes to the respiratory tissue (142, 143). Together, this implies that TLR4 is required to promote an inflammatory response that will induce ALI and damage respiratory tissue. If TLR4 is pathogenic in ALI, then TLR4 inhibitors may be a beneficial therapeutic to treat this disease. In a recent study, mice injected with the TLR4 antagonist FP12 exhibited protection against influenza-induced ALI (57). Other more novel technologies have recently been used to inhibit TLR4 expression and attenuate ALI in mice. A small interference RNA (SiRNA) which targets and silences TLR4 through neutrophil coated nanoparticle delivery was utilized to improve ALI in an LPS-stimulated mouse model. TLR4 protein and downstream products of TLR4 signaling including IL-1β and TNF-α, were reduced in the SiRNA-treated mice when compared to the placebo group. Moreover, there was no observed toxicity after 18 days of stimulation (144). Therefore, these more novel therapeutics may be a promising area of investigation for the targeted treatment of ALI. As ALI is complex and etiologies vary, various bacterial and viral PAMPs including *S. pneumoniae, S. aureus*, COVID-19 and seasonal Influenza (145). Some endogenous DAMPs have also been associated with ALI pathogenesis. In renal ischemia reperfusion injury and hemorrhage induced ALI in mice models, the inhibition of HMGB1 has demonstrated to ameliorate ALI (146, 147). Additionally, radiolabeled HA fragments have been demonstrated to be a potentially useful biomarker in mice for the detection of ALI (148). Therefore, measurement of TLR4-binding DAMPs may represent a potential biomarker for ALI and inhibition of ligands HMGB1, or HA could be explored as novel therapeutic strategies to treat ALI.

There is opposing evidence that TLR4 is decreased in ALI, and induction of this receptor could provide protective effects. One study found through quantitative real-time reverse PCR (qRt-PCR) that TLR4 mRNA was decreased in PBMCs of patients with ALI when compared to matched controls. This decrease in TLR4 expression was associated with higher mortality, as patients with higher TLR4 expression survived on average 5 days longer than those with low TLR4 expression (149). The correlation between reduced TLR4 expression and increased mortality suggests that TLR4 may play a protective role in ALI, and that TLR4 dysfunction can lead to a dampened homeostatic process. Similarly, TLR4 KO ALI mice models showed an increase of NF-κβ and p38/Glyceraldehyde-3-phosphate dehydrogenase (GAPDH) when compared to the ALI WT mice with an active TLR4 (150). This indicates that expression of NF-κβ and p38/GAPDH can be amplified and induce pro-inflammatory responses and cell death without the need for TLR4 expression. Additionally, TLR4 KO mice treated with low molecular weight HA had markedly increased IL-1β, IL-6 and TNF-α in bronchoalveolar lavage fluid (BALF). TLR4 KO mice also displayed severe lung injury when compared to WT controls, with TLR4 anti-inflammatory effects thought to be MyD88 dependent (151). This indicates that normal TLR4 expression may regulate chronic inflammation and decrease the chance of severe lung injury. Overall, this data suggests a functional TLR4 receptor may prevent oxidative stress and is required to resolve inflammation and prevent severe injury in an ALI context.

There is conflicting evidence regarding the role of TLR4 in ALI. Some reports suggest an upregulation of TLR4 may lead to more severe disease, whilst others suggest TLR4 plays a protective role (135, 136, 149). ALI is a heterogenous disease that can be caused by varying etiologies ranging from direct causes (e.g. infection with viral, bacterial and other pathogens) to indirect causes (e.g. non-thoracic trauma, pancreatitis and severe burns). Therefore, there is a need for further investigation into potential therapeutics for these divergent subtypes of ALI.

#### 3.2.2 Chronic obstructive pulmonary disease

Chronic obstructive pulmonary disease (COPD) is a progressive disease of which tobacco smoking is the primary risk factor. The treatment for COPD mainly focuses on clearing the airways with bronchodilators together with intermittent inhaled and/or oral corticosteroids for disease flares and antimicrobials for infective exacerbations (152).

There is some evidence to support the association of TLR4 polymorphisms with COPD development. In Greece, a cohort of over 100 patients with COPD found that the TLR4 rs4986791 polymorphism increased the risk of COPD over 2-fold when compared to smokers without COPD (153). Additionally, a study of over 150 COPD patients in China found that TLR4 rs10759932 and TLR4 rs2737190 exhibited an increased risk for COPD when compared to over 200 age and sex-matched healthy controls (154). In an investigation of over 350 COPD patients who were smokers, it was found that TLR4 rs4986790 A/A genotype was associated with an increased COPD risk in smokers when compared to healthy carriers with the A/G or G/G genotypes (155). Furthermore, a study conducted in China found that the TLR4 rs10759932, rs2737190, rs7873784, rs11536889 and rs10983755 SNPs in 150 COPD patients were associated with a decreased risk of COPD when compared to over 1,700 healthy controls (156). Therefore, TLR4 rs4986791 and rs10759932 could potentially increase the risk of COPD, with some genetic variants in rs4986790 also representing an increased COPD risk. While other SNPs like TLR4 rs4986790, rs10759932, rs7873784, rs11536889 and rs10983755 may decrease risk. The exact role of rs4986790 should be investigated further due to the conflicting evidence of COPD risk.

TLR4 has been implicated in the development of COPD in animal models and human studies. TLR4 mRNA and protein were increased in mouse models of COPD after cigarette smoke exposure. Post cigarette smoke exposure, TLR4 KO mice had a decreased inflammatory response signified by a decline in collagen deposits when compared to cigarette smoke exposed WT controls (157). A pilot study of endobronchial biopsies from a cohort of 8 patients who smoked had a significant increase in CD8^+^TLR4^+^ T cells in the lungs when compared to healthy matched controls (158). Notably, over 90% of CD8^+^ T cells in the lungs of COPD patients expressed TLR4, when compared to almost 20% in healthy volunteers. This upregulation was thought to be a function of high cigarette smoke exposure which was confirmed using *in vitro* cultures of isolated CD8^+^ T cells challenged with cigarette smoke condensate (158). Similarly, BALF samples from ICU patients with COPD secondary to smoking had a larger population of TLR4^+^ cells, which were predominantly neutrophils, when compared to the non-smoking and smoking without COPD groups (159). In COPD patients, TLR4 expression has been reported to be increased on CD8^+^ T cells and neutrophils, suggesting a significant pro-inflammatory response spanning innate and adaptive immunity. This could represent a potential avenue for novel targeted therapeutics for COPD. The TLR4 antagonist TAK-242 has shown to reduce smoking-induced respiratory inflammation in mice by reducing TLR4 and downstream cytokine expression (160). More recently, low doses of naltrexone in mouse models of COPD reduced oxidative stress through the inactivation of the TLR4 pathway (161). Therefore, TLR4 inhibitors could be an efficacious therapeutic to ameliorate hyperactive immune responses exhibited in COPD. TLR4-binding DAMPs have also been associated with COPD pathogenesis. In mice, HMGB1 exacerbates COPD by encouraging macrophage class switching the pro-inflammatory M1 phenotype (162). Additionally, in bronchoalveolar lavage (BAL) samples from patients with acute exacerbations of COPD, it was shown that HS as well as Chondroitin Sulphate (CS) were elevated when compared to controls without COPD (163). Furthermore, HA synthase 2 dysfunction in COPD increases airway inflammation and neutrophil recruitment (164). This indicates that HA may play a protective role in COPD whilst HMGB1 may exacerbate disease.

### 3.3 Cardiovascular diseases

#### 3.3.1 Coronary artery disease

Coronary artery disease (CAD) involves the narrowing of arteries and subsequent inflammation of arteriole tissue, threatening blood supply to the myocardium. Current treatment options for CAD commonly involve a combination of diet and lifestyle changes as well as the implementation of antithrombotic/antiplatelet treatments, surgery to introduce arterial stents to widen ischemic arteries or coronary artery bypass graft surgery (165).

The role of the TLR4 pathway and associated polymorphisms in CAD development has not been well established. A meta-analysis of almost 14,000 patients found that the TLR4 rs4986791 polymorphism was associated with reduced CAD risk in studies from China, but not in studies conducted in Europe and the USA (166). Further research is warranted at a larger global scale to determine the risk for CAD associated with this polymorphism.

Increased TLR4 expression may propagate CAD risk. A study of mRNA expression in coronary plaque debris from patients with acute coronary syndrome and stable angina found that TLR4 mRNA was more highly expressed in the acute coronary syndrome cohort than stable angina group (167). This suggests that TLR4 expression may drive CAD pathogenesis and promote plaque formation. Furthermore, certain transcription factors and proteins have been associated with a decreased risk of CAD through a reduction of TLR4 expression. For instance, regulatory factor X1 (RFX1), a gene that regulates proliferation, was decreased in monocytes of patients with CAD, whilst TLR4 was significantly increased in this cohort. When RFXI was knocked out, TLR4 was overexpressed, whereas when RFX1 expression increased, TLR4 expression decreased (168). This highlights that RFX1 may play a regulatory role in decreasing TLR4 expression and preventing CAD. Similarly, a more recent study found that serum proprotein convertase subtilisin/kexin-type 9 (PCSK9), which regulates cholesterol, was negatively correlated with serum TLR4 and DAMP HMGB1 expression in CAD patients with statin prescriptions. PCSK9 was positively correlated with TLR4 in the control group, suggesting PCSK9 may play a role in preventing CAD (169). Although it appears TLR4 may play a pathogenic role in CAD, there has been little investigation into TLR4 antagonists. One mouse study that modelled type 2 diabetes found that the TLR4 antagonist *Rhodobacter* sp*haeroides* LPS (Rs-LPS) successfully downregulated TLR4 expression on monocytes and attenuated atherogenesis (170). TLR4 appears to drive atherogenesis and plays a pathogenic role in CAD and the development of atherosclerosis. Further delineation into the TLR4 pathway in CAD could lead to the development of targeted TLR4 therapeutics for this disease. DAMPs have also been associated with the formation of atherosclerosis and CAD. HS is thought to induce atherosclerotic plaque formation by clustering in subendothelial tissue. A monoclonal antibody for a proliferation-inducing ligand (APRIL) has been shown to directly bind to HS and protect against CAD by reducing plaque formation in mice on an atherogenic diet (171). Furthermore, HMGB1 has been associated with non-calcified plaque formation in CAD patients with stable atherosclerosis (172). Therefore, HS and HMGB1 may also be effective therapeutics for CAD.

#### 3.3.2 Myocardial infarction

Globally, myocardial infarction (MI) is a leading cause of mortality and can arise from pre-existing CAD (173, 174). Timely coronary reperfusion is required for improvement post-MI and includes treatments such as fibrinolysis and primary percutaneous coronary intervention (PCI) (175). Investigations into more effective therapeutics could pave the way for improved tertiary prevention post-MI.

TLR4 has been implicated in the pathogenesis and development of MI. TLR4 is reported to be upregulated in MI patients, however no genetic variant in the rs4986790 polymorphism has been associated with the risk of MI from a meta-analysis of over 15,000 patients (176, 177). One paper reported that TLR4 was highly expressed on the platelets of 40 patients with MI following LPS stimulation when compared with platelets from 40 controls with no cardiovascular disease (177). Similarly, another study that involved stimulating monocytes with LPS found an increase of TLR4–24 hours post MI occurrence. TLR4 mRNA and protein levels, as well as downstream cytokines TNF-α and IL-1β were upregulated in MI patients compared to healthy volunteers, which was associated with increased risk of heart failure (178). Additionally, a mouse model of chronic heart failure following MI showed that TLR4 expression was increased and sensitized on cardiomyocytes and had an increased level of TLR4 mRNA and protein 4 weeks post MI (179). Interestingly, this data implies that cardiomyocytes exhibit some innate immune response as TLR4 activation on cardiomyocytes led to a proinflammatory response through increased TNF-α and IL-6 post MI. TLR4 has been proposed as a potential biomarker for recurrent MI. In a study conducted in elderly patients (65–88 years old) with coronary stents it was found that TLR4 was upregulated on the PBMC’s of patients with recurrent MI, when compared to patients who had received coronary stents but did not have recurrent MI (180).

TLR4 inhibition may be a potential area of research for the improvement of MI management. The TLR4 antagonist TAK-242 reduces cardiac inflammation by reducing IL-6, IL-1β and TNF-α and reduction of fibrosis in mouse models of MI (181). TLR4 binding DAMPs appear to also play a role in MI pathogenesis. In over 200 ST-segment-elevation MI patients the plasma levels of high molecular weight HA was significantly lower when compared to healthy controls (182). Furthermore, elevated circulating HGMB1 in patients 4 months post-MI promoted pro-inflammatory M1 macrophage polarization and induced NLRP3 inflammasome signaling (183). In rats, HS also potentiates fibrosis post-MI by activating the TLR4 pathway and driving ICAM-1 and vascular cell adhesion molecule 1 (VCAM-1) production which facilitates cardiac fibroblast leukocyte adhesion. An siRNA against ICAM-1 and VCAM-1 prevented fibrosis driven by HMGB1/TLR4 binding (184). Therefore, HMGB1 and HS in addition to TLR4 could be potential therapeutic targets to ameliorate MI. Further research into TLR4 specific and TLR4 ligand inhibitors is needed in human studies to fully understand the effects of TLR4 inhibition in MI patients.

### 3.4 Autoimmune disorders

#### 3.4.1 Inflammatory bowel disease

Inflammatory bowel disease (IBD) is comprised of several conditions with the major forms being Crohn’s disease and Ulcerative Colitis. IBD is characterized by severe chronic inflammation of the digestive system which can lead to mucosal tissue damage and tumorigenesis. Treatment for IBD includes the use of corticosteroids, aminosalicylates, and invasive surgical treatment to alleviate IBD-related symptoms, as well as immunomodulating therapeutics such as TNF-α inhibitors and the anti-integrin monoclonal antibody vedolizumab (185, 186).

TLR4 SNPs have been significantly associated with IBD risk. In a meta-analysis of almost 50 studies, it was shown that the TLR4 rs4986790 and rs4986791 SNPs were significantly associated with IBD in IBD patient cohorts when compared to the controls (187). Therefore, TLR4 SNPs such as rs4986790 and rs4986791 may be a genetic determinant of IBD.

TLR4 expression has also been implicated in IBD pathogenesis. One study found that activation of TLR4 in the intestinal mucosa, drives dual oxidase 2 (DUOX2) transcription, leading to the production of hydrogen peroxide which promotes tumorigenesis (188). This suggests that TLR4 could assisting in promoting progression of malignancies that are associated with severe IBD such as colorectal cancer (189). In one study, 16S rRNA sequencing was conducted on fecal samples extracted from Dextran sulfate sodium salt induced colitis TLR4 KO and WT mice. This was associated with an increased presence of various bacterial species including *Proteobacteria*, *Tenerlcutes* and *Deferribacteres* which increased gastrointestinal bleeding and anemia in TLR4 KO mice when compared to the WT controls (190). This data suggests that TLR4 may play a protective role in IBD by preventing bacterial infiltration in the digestive tract and reducing disease flares. Furthermore, a recent study found that the TLR4 antagonists TAK-242 and IAXO-102 failed to suppress excessive immune responses seen in intestinal inflammation in mouse models of IBD (191). Although there is conflicting evidence of the role of TLR4 in IBD, one study showed that the synthetic TLR4 antagonist, CRX-526 reduced intestinal inflammation in mouse models of IBD (62). Although IAXO-102 and TAK-242 were found to not be effective, CRX-526 appeared to exhibit protective effects, which may be due to the differences in mechanisms of action. TAK-242 acts by binding to the intracellular domain of TLR4 and inhibiting TLR4 interaction between its adaptor proteins such as TRAM and TIRAP whereas IAXO-102 modulates interaction between TLR4 and its co-receptors such as MD2 and CD14 (192, 193). CRX-526, unlike TAK-242 and IAXO-102 is a competitive antagonist for TLR4 and therefore directly competes with binding at the TLR4 binding site (62). As IBD can be associated with an increase of pathogenic bacteria, a more competitive binding antagonist to LPS such as CRX-526 may be more effective than TAK-242 and IAXO-102. Therefore, future studies should compare the effect of competitive antagonists such as CRX-526 with other antagonists like TAK-242 and IAXO-102. TLR4-binding PAMPs and DAMPs have also both been implicated in IBD pathogenesis. IBD is characterized by microbiota dysbiosis and has been associated with increased levels of various bacteria including *Escherichia coli, Bacteroides fragilis, Fusobacterium varium and Campylobacter concisus (*194). DAMPs have also been associated with IBD pathogenesis. In patients with IBD, HMGB1 mRNA has been found to be elevated in inflamed colonic tissue acquired from biopsy when compared to tissue that was not inflamed (195). Furthermore, the use of anti-HMGB1 in IBD induced mice resulted in a downregulation of proinflammatory M1 macrophages and TLR4 expression as well as reduced mucosal damage when compared to IBD induced mice that did not receive anti-HMGB1 treatment (195). Excessive production of HA in IBD patients has also been associated with non-responsiveness of monoclonal antibodies such as vedolizumab and infliximab (196). Therefore, inhibiting DAMPs such as HMGB1 and HA could improve IBD symptoms and reduce gastrointestinal inflammation.

#### 3.4.2 Diabetes mellitus type 1

Diabetes mellitus type 1 (T1D) is an autoimmune disease characterized by the destruction of pancreatic β cells affecting the pancreases’ ability to produce insulin. T1D usually manifests in childhood and the main treatment for T1D focuses on insulin replacement (197).

TLR4 polymorphisms have been studied in relation to T1D development, however the TLR4 polymorphisms, namely, rs4986790, rs4986791 and rs5030719 were not associated with T1D in a European cohort (198). Further larger global investigations should be conducted to determine genetic risk.

The TLR4 signaling pathway has been implicated in T1D pathogenesis. One study found that in pediatric patients with newly diagnosed (<3 months post diagnosis) and chronic T1D (>3 months post diagnosis), there was an increased expression of TLR4 on CD8^+^ and CD4^+^ T cells, as well as CD19^+^ B cells when compared to controls (199). In contrast, another study which utilized hyperinsulinemia (HI) and hyperglycaemia (HG) clamps to provide an infusion to maintain glucose and insulin levels were studied in T1D and healthy controls to determine risk of cardiovascular events (200). Whole blood taken from T1D patients showed that this cohort had an almost 50% decreased expression of TLR4 after 24 hours of HI/HG infusion when compared to the controls. However, whole blood taken from healthy controls showed a 6-fold increase in TLR4 expression as well as tissue factor-procoagulant activity (TF-PCA) expression 2–4 hours after infusion, whilst T1D patients showed a significant decline in both parameters (200). This implies that TLR4 expression is dysregulated in T1D and may exhibit immune tolerance, as HI/HG infusion in healthy controls leads to an increased TLR4 expression. Additionally, it has been suggested that TLR4 plays a protective role in T1D, with one study showing 10-week-old non-obese diabetes (NOD) mice who were TLR4 KO had an accelerated development of T1D, manifesting 59 days earlier than WT TLR4^+^ controls (201). This implies that functional TLR4 expression could assist in preventing T1D development.

TLR4 disease modifying drugs have been proposed as therapeutics for T1D. 2-month-old NOD mice treated with TAK-242, a selective TLR4 inhibitor, displayed decreased CD4^+^ T cell activation and inhibited auto-antigen specific proliferation as well as a delayed T1D onset by approximately 6–8 weeks when compared to the untreated NOD mice (202). This implies that inhibition of TLR4 could ameliorate autoimmune responses in early T1D development by targeting auto-reactive CD4^+^ T cells and subsequently preventing infiltrative insulitis. Contrary, a study of NOD mice stimulated with a monoclonal anti-TLR4/MD2 showed that weight and insulin positive islet cells increased whilst blood glucose levels significantly decreased. Furthermore, this study demonstrated that the induction of CD11b^+^Ly6G^+^ myeloid cells from TLR4 activation led to a decrease in blood glucose (203). TAK-242 may therefore be effective in preventing T1D development by blocking TLR4, however when T1D progresses, TLR4 agonistic activity may promote the induction of protective immune cells such as CD11b^+^Ly6G^+^ myeloid cells. Furthermore, TLR4 binding DAMPs have been associated with T1D pathogenesis and could be an area of therapeutic targeting to block pathogenic autoreactivity. In NOD mice, elevated HMGB1 expression was observed in pancreatic islet cells when compared to mice without T1D (204). Additionally, 65% of NOD mice with early T1D that received anti-HMGB1 treatment experienced T1D remission by alleviating T_reg_ dysfunction (205). Therefore, inhibition of HMGB1 in early stages of T1D could potentially inhibit disease progression. Interestingly however, it is thought that other DAMPs may play a protective role in T1D. HS has been associated with the survival of pancreatic β cells, with degradation of HS through the HS degrading enzyme heparanase being associated with β cell death. Treatment with a heparanase inhibitor named PI-88 protected approximately 50% of NOD mice from T1D (206). Therefore, inhibiting heparanase may be a therapeutic option for T1D.

#### 3.4.3 Systemic lupus erythematosus

Systemic lupus erythematosus (SLE) is an autoimmune disease which is characterized by dysregulated activation of immune cells and autoantibodies associated with tissue and organ destruction of the skin, kidneys, musculoskeletal and central nervous system (207). Currently SLE is treated based on the disease severity and organs affected and can vary from sun avoidance, conventional synthetic disease modifying immunosuppression and more targeted monoclonal antibody therapies such as anti-CD20 and anti-APRIL/B Lymphocyte Stimulator (BlyS) (207).

There is no compelling evidence that TLR4 polymorphisms influence the development or risk of SLE. Of note, both meta-analyses conducted in 2016 and 2024 found no association between TLR4 rs4986790 and TLR4 rs4986791 polymorphisms and risk of SLE (208, 209).

Despite the lack of evidence regarding the genetic association of TLR4 SNPs in SLE, the expression and regulation of TLR4 transcript and protein have been implicated in SLE development. Levels of TLR4 mRNA were higher in a study of 70 SLE patients as compared to 34 healthy controls (210). mRNA and immunohistostaining showed an upregulation of sTLR4 expression in saliva, renal and skin biopsies of patients with SLE when compared to non-SLE patient and non-renal disease controls (211, 212). The anti-double stranded DNA (anti-dsDNA) auto-antibodies central to SLE diagnosis, have been shown to bind to TLR4 on macrophages and induce ROS, as well as induce the production of caspase-1 in mouse models and patients (213). In a mouse model of SLE, TLR4 and downstream products IRF3 and NF-κβ were significantly increased in SLE as compared to healthy control mice (214). Additionally, it has been suggested that somatic nuclear autoantigenic sperm protein (sNASP), a histone chaperone that contributes to autoimmunity and disease progression, drives the overactivation of the TLR4/TAK-1 pathway through TRAF6 modulation (215). The binding of HMGB1 to TLR4 in glomerular endothelial cells has also been associated with nephrotic injury in SLE when compared to matched healthy controls, implying that TLR4 activation is necessary for SLE induced nephritis (216). Although it appears that TLR4 may play a pathogenic role in SLE, no TLR4 antagonists have reportedly been tested in models of SLE. However, TLR4-binding DAMPs have been associated with SLE pathogenesis and could also be targets for therapy. Circulating HMGB1 has been found to be highly expressed in SLE patients, and it has been postulated through an SLE mice model that HMGB1 drives inflammation in SLE by activating dendritic cells and inducing T cell response (217). It has also been demonstrated through *in vitro* cultures of kidneys from SLE patients with nephritis that HA expression is associated with tissue destruction when compared to normal kidney tissue extracted from renal carcinoma patients (218). When inhibiting HA synthesis through a HA inhibitor (hymecromone) in SLE mice, it was shown that tissue damage was decreased when compared to mice who did not receive treatment (218). Therefore, inhibiting HMGB1 and HA could be a therapeutic option for SLE treatment.

### 3.5 Metabolic diseases

#### 3.5.1 Obesity and related illnesses

Obesity is an increasingly prevalent and heterogeneous disease that can increase susceptibility to various other conditions such as T2D, cardiovascular disease and certain cancers (219–221). It is characterized by chronic low-grade inflammation which contributes to subsequent insulin resistance. Treatment of obesity generally consists of lifestyle and diet changes. However more recently, disease modifying agents such as glucose-dependent insulino-tropic polypeptide (GIP) and glucagon-like peptide-1 (GLP-1) receptor agonists such as Tirzepatide and Semaglutide have been widely repurposed for weight loss (222, 223).

Obesity and insulin resistance have genetic risk factors which could be associated with TLR4 SNPs. A pilot study of 100 obese patients in Mexico found that TLR4 rs10983755, rs4986791 and rs4986790 SNPs positively correlated with obesity and elevated steatosis in the cohort with obesity when compared to 104 average weight controls, with these genes being related to a dysfunction in TLR4 expression and activation (224). Moreover, a study of 205 females in Qatar, demonstrated that obesity was significantly associated with TLR4 rs4986790 and rs4986791 SNPs, alongside markedly increased TLR4, IL-10 and IL-6 protein expression (225). TLR4 genetic variants may therefore play a key role in obesity development.

There have been several reports in the last decade investigating the role of TLR4 in the development of obesity and obesity-related illnesses. One mouse study proposed that saturated free fatty acids, combined with S100A9 protein expression induces the production of TLR4 which induces a feedback loop through IL-1β activation in macrophages, and creates S100A9 overexpression in obesity (226). However, another mouse study focusing on high-sugar diets suggested that S100A9 is downregulated on mononuclear cells in the bone marrow and liver and stimulates dysfunctional macrophage activation through the TLR4 pathway (227). Therefore, the exact relationship between S100A9 and TLR4 remains controversial. There is growing evidence that TLR4 is strongly associated with the development of obesity, such as production of TLR4 downstream molecules IL-1β, IL-6 and TNF-α in adipose tissue of mouse models of obesity (228). Additionally, the TLR4-binding DAMP HMGB1 is increased in obese patients with normal glucose as well as patients with T2D (229). In TLR4 KO mice, steatosis in hepatocytes, and liver and adipose inflammation were decreased. In these TLR4 KO mice triglycerides, cholesterol, TNF-α, IL-6 and IL-1β were all significantly lower as compared to heterozygous TLR4^-/+^ mice (230). A recent study further characterized this finding by showing that TLR4 KO mice reprogrammed hepatocytes by suppressing oxygen consumption rate and therefore reducing ROS development (231). Therefore, hyperactive TLR4 expression on hepatocytes could contribute to insulin resistance and fatty liver by increasing inflammation and ROS in the liver and adipose tissue as well as propagating steatosis in hepatocytes. TLR4 has also shown to be implicated in the pancreas during T2D. Excessive TLR4 activation can induce β cell failure by blocking the replication of β cells, leading to the progression of obesity related diabetes (232, 233). Other studies suggest that TLR4 LOF could prevent insulin resistance and play a protective role against cardiovascular disease when exposed to high fat diets (234, 235). Furthermore, GLP-1 receptor agonists have shown to significantly reduce TNF-α expression in not only circulating PBMC’s but also in the lungs and spleen of mice stimulated with LPS, indicating the heightened TLR4 expression in obesity can be attenuated with current GLP-1 therapies (236).

Chronic and systemic inflammation is the hallmark for obesity. Therefore, TLR4 antagonists could be a potential treatment alternative. TAK-242 has shown to exert not just a reduction in adipocytes but also exhibited neuroprotective effects in mice models of obesity (237). Furthermore, TAK-242 as well as Eritoran tetrasodium (E5564) improved insulin production and reduced hepatic glucose production in rat models (238). However, Eritoran (E5564) administration in a small double-blind RCT of 9 obese patients (BMI 30-37) and healthy controls (BMI <25) did not confer a protective role against insulin resistance (7). This could be a result of the small sample size utilized and differing physiology between rats and humans. Further *in vitro* and *in vivo* studies of both TAK-242 and Eritoran is warranted to investigate if TLR4 antagonists may improve chronic inflammation characterized in obesity and ameliorate insulin resistance. DAMPs have also been associated with obesity and obesity-related illnesses. In T2D patients circulating HA levels were increased when compared to healthy controls and T1D patients (239). Furthermore, 33 patients with obesity (>29.9 BMI) had increased circulating fragments of low molecular weight HA when compared to patients with a BMI below 29.9 (240). Additionally, plasma HMGB1 has been significantly positively correlated with higher BMI and waist circumference (241). This indicates that both HA and HMGB1 may be key pro-inflammatory drivers in obesity. Interestingly however HS has been associated with differentiating white adipose tissue and maintaining glucose tolerance in mice (242). Therefore, whilst HA and HMGB1 may propagate inflammation in obesity, HS could potentially ameliorate inflammation in this disease. Therapeutic targeting of TLR4-binding DAMPs such as HA, HMGB1 and HS should be investigated for the treatment of obesity driven inflammation.

#### 3.5.2 Pancreatitis

Pancreatitis is characterized by inflammation of the pancreas due to various risk factors such as excessive alcohol consumption, gallstones and hyper-triglycerides, and can be acute or chronic in nature. It has been estimated that 20-30% of patients with pancreatitis develop severe disease that requires organ support in an ICU (243, 244). The current mainstay of treatment is supportive care, with no known disease modifying agents. Therefore, the introduction of therapeutics which target immune responses prevalent in pancreatitis may assist in decreasing the number of patients that develop severe pancreatitis (243).

There is conflicting data regarding the role of TLR4 polymorphisms in pancreatitis development and severity. A study that investigated 100 patients with acute pancreatitis found that TLR4 rs4986791 and rs4986790 SNPs increased susceptibility for acute pancreatitis when compared to 101 healthy volunteers with no pancreatitis, gallstones or other risk factors for acute pancreatitis (245). Furthermore, when looking at 108 patients with acute non-biliary pancreatitis, the A/G genotype of the TLR4 rs4986790 was associated with a higher leukocyte count when compared to the A/A genotype. However, this was not associated with the overall prognosis and clinical outcome (246). Conversely, other studies suggest there is no association between TLR4 SNPs and pancreatitis. A meta-analysis of 1255 acute pancreatitis found no association between the TLR4 rs4986791 and rs4986790 and acute pancreatitis risk when compared to 998 controls (247). The association between pancreatitis and TLR4 SNP’s is unclear, and further investigation is required to determine genetic risk.

Increased TLR4 expression has been implicated in the development of pancreatitis. During the early stages of mild acute pancreatitis, TLR4 is highly expressed on monocytes in human PBMCs (248). Mouse models of severe acute pancreatitis have demonstrated that the levels of the TLR4-binding DAMP HMGB1, TLR4 and associated downstream molecules on monocytes were elevated, indicating that TLR4 may be important in the progression of pancreatitis and could be mediated by HMGB1 binding (249). HMGB1 has also been associated with rat models of chronic pancreatitis, with HMGB1 binding to TLR4 and driving pancreatic fibrosis through pancreatic stellate cell activation and extracellular matrix deposition, when compared to rats with moderate acute pancreatitis (250). Therefore, in mild acute pancreatitis, increased TLR4 activation appears to resolve, while ongoing activation of TLR4 may promote disease severity and progression in chronic disease. TLR4 deficiency in mouse models exacerbated injury to intestinal and pancreatitis tissue due to an imbalance in the gut microbiome and subsequent Paneth cell dysfunction. These cells which are found in the small intestine’s epithelium are crucial for immune defense as they secrete protective antimicrobial peptides for the gut microbiome (251).

Various compounds have shown to reduce pancreatitis severity by inhibiting the TLR4 pathway. The antimalarial Artesunate exerted protective effects in mouse models of LPS-induced pancreatitis, potentially by inhibiting the TLR4 pathway and attenuating pancreatic inflammation (252). TAK-242 administration in high-fat diet-induced pancreatitis rat models demonstrated that TLR4 inhibition attenuated structural pancreatic damage and necroptosis (253). TAK-242 has also shown to be effective in rat models of chronic pancreatitis by reducing apoptosis of acinar cells as well as attenuating weight loss associated with pancreatitis (254). Furthermore, a novel synthetic peptide consisting of 15 amino acids called HTD4010 ameliorated pancreatic tissue damage in mouse models of acute biliary pancreatitis by inhibiting the TLR4 pathway when compared to WT mice with acute biliary pancreatitis (255). These studies suggest that TLR4 inhibition ameliorates pancreatitis and reduces ROS production in animal models, however this has not been translated into human trials. Therefore, utilizing TLR4-targeted therapeutics for pancreatitis could be a potential area of research. Additionally, DAMPs which bind to TLR4 have been associated with pancreatitis pathogenesis. In a mouse model of acute pancreatitis, it was demonstrated that the cleavage of HS worsens pancreatitis by inducing neutrophil infiltration and activating the NF-κβ pathway. Therefore, HS could play a protective role in remodeling pancreatic tissue and inhibiting heparanase may be a potential therapeutic target for acute pancreatitis (256). Although HS may play a protective role in pancreatitis, HMGB1 has been associated with driving fibrosis in acute pancreatitis and progressing to chronic pancreatitis in rats (250). Therefore, DAMP-targeted therapeutics such as a HMGB1 antagonist or a HS agonist may ameliorate pancreatitis.

## 4 Conclusion & future directions

Canonically, TLR4 plays an important role during bacterial infection to develop a pro-inflammatory response which targets and helps to eliminate pathogens. However, the function of TLR4 may be more complex than simply an innate receptor which initiates the first line of defense. TLR4 may also play a role in more adaptive pathways and chronic immune responses seen in autoimmune and chronic disease which requires further investigation. Currently, there are no TLR4 specific modulating therapeutics available for clinical use. Existing TLR4 agonists have showed efficacy as vaccine adjuvants in infectious diseases. GLA-SE and GSK1795091 have also been effectively repurposed for the treatment of cancers in clinical trials (48, 51). Although these agonists have showed efficacy as adjuvants in infectious disease vaccines and for the treatment of cancer, little research has been conducted into the efficacy of these drugs in further inflammatory diseases. As TLR4 activation can induce pro-inflammatory responses which can lead to toxicity, it is important to carefully design the therapeutic dosage for the treatment of inflammatory diseases. Some studies suggest that TLR4 has a dampened response in AD, MS, PD, ALI, IBD and T1D and therefore TLR4 agonists may provide a therapeutic benefit. However, further *in vitro* and *in vivo* work would need to be conducted to determine the biological differences in early and later stages of these diseases, as well as effective and safe therapeutic dosage. For instance, in PD, it appears that TLR4 may be hyperresponsive at the early stages of disease, whereas in later stages the receptor is dampened. TLR4 antagonists have demonstrated efficacy in models of influenza, sepsis, vascular inflammation, diabetic nephropathy and IBD but have not yet been translated to clinical trials. In diseases where TLR4 appears predominantly pathogenic, such as AD, MS, COPD, CAD, MI, SLE, obesity and pancreatitis; TLR4 inhibitors may provide effective protection against chronic inflammation. Currently, the diseases that have the strongest indication for TLR4 inhibitors include AD, MS, pancreatitis and obesity, where at least two different TLR4 inhibitors have been tested and shown efficacy in animal and *in vitro* models of these diseases. Further *in vitro* and *in vivo* studies which delineate the exact nature of TLR4 in each disease would be beneficial to determine whether TLR4 agonists or antagonists could be a potential treatment option. Pre-clinical studies in mice and non-human primates will be important to test the implications of TLR4 agonists and antagonists. *In vivo* modulation of TLR4 is a compelling prospect for future precision medicine for a potentially wide variety of autoimmune and inflammatory conditions. Other mediators including DAMP targeting or downstream targeting of inflammasomes or cytokines may also be potential therapeutics. Targeting DAMPs such as HS, HMGB1 and HA directly or through biologics; or targeting enzymes which cleave these DAMPs such as heparanase have demonstrated to be potential options in all the diseases mentioned throughout this article. Other potential strategies include inhibiting the NLRP3 inflammasome pathway through mediators such as MCC950, which targets non-canonical activation of NLRP3 through caspase-4/5 (257). Anakinra which has been utilized to treat inflammatory diseases could be a potential treatment option as it can bind to IL-1β and can inhibit the NLRP3 inflammasome. For example, the combination of anakinra and MCC950 ameliorated ALI by blocking the NLRP3 inflammasome in ALI induced mice (258). MCC950 has been utilized in several pre-clinical trials, and has demonstrated efficacy in reducing severe disease, however a phase II clinical trial for rheumatoid arthritis observed MCC950 induced hepatotoxicity (259). Therefore, further investigation should be conducted into MCC950 safety and tolerance. Although so far TLR4 inhibitors have not been efficacious in clinical trials, there is an expanding understanding of TLR4 in human disease as well as an array of therapeutic targets that could be explored to decrease inflammation. Further research should be conducted into both direct and indirect TLR4 modulators for candidate human diseases where TLR4 has consistently been shown to be dysregulated.
